# Parasite Diversity in a Freshwater Ecosystem

**DOI:** 10.3390/microorganisms11081940

**Published:** 2023-07-29

**Authors:** Amana Shafiq, Farzana Abbas, Muhammad Hafeez-ur-Rehman, Bushra Nisar Khan, Ayesha Aihetasham, Iffat Amin, Hmidullah, Ramzi A. Mothana, Mohammed S. Alharbi, Imran Khan, Atif Ali Khan Khalil, Bashir Ahmad, Nimra Mubeen, Muneeba Akram

**Affiliations:** 1 Department of Fisheries and Aquaculture, University of Veterinary and Animal Sciences, Lahore 54000, Pakistan; mhafeezurehman@uvas.edu.pk (M.H.-u.-R.);; 2 Institite of Zoology, University of Punjab, Lahore 54590, Pakistan; 3 Department of Zoology, Kinnaird College for Women, Lahore 54000, Pakistan; 4 Department of Zoology, Islamia University Bahawalpur (Bahawalnagar Campus), Bahawalnagar 63100, Pakistan; 5 Department of Pharmacognosy, College of Pharmacy, King Saud University, Riyadh 11451, Saudi Arabia; 6 Department of Biochemical Engineering, College of Engineering, Keimyung University, 1095 Dalgubeol-Daero, Dalseo-gu, Daegu 42601, Republic of Korea; 7 Department of Pharmacognosy, Faculty of Pharmaceutical and Allied Health Sciences, Lahore College for Women University, Lahore 54000, Pakistan; 8 Department of Zoology, University of Malakand, Chakdara 18800, Pakistan

**Keywords:** protozoa, monogeneans, trematodes, prevalence of infection, mean density, diversity

## Abstract

Parasites are a significant component of biodiversity. They negatively affect fish appearance, growth, and reproduction. In this study, the prevalence of infection, diversity, and mean intensity of parasites were examined in 9 freshwater fish species (45 samples per fish species). Ecto-parasites were examined on the skin, gills, and fins with a hand lens. Wet mounts were prepared using mucosal scrapings from all the external and internal organs of the sampled fish. Microscopy, muscle compression, and the pepsin-HCL artificial digestion technique were also performed. In this study, 26 species of parasites were identified including three taxa belonging to 9 species of protozoan parasites, 11 treamtodes, and 6 monogenean parasites. The identified protozoan parasites were *Entamoeba histolitica*, *Chilodonella* sp., *Coccidia* sp., *Costia* sp., *Cryptobia* sp., *Ichthyopthiris-multifilis*, *Microsporidia*, *Piscinoodinium* sp., and *Ichthyobodo necator*. The identified trematode parasites were *Fasciola gigantica*, *Echinostoma revolutum*, *Fasciola hepatica*, *Haplorchis pumilio*, *Brachylaima cribbi*, *Echinostoma cinetorchis*, *Neascus* sp., *Deropegus* sp., *Trematode Soldier*, *Centrocestus formosanus*, and *Clinostomum marginatum.* The identified monogenean parasites were *Dactylogyrus limipopoensis*, *Dactylogyrus anchoratus*, *Dactylogyrus myersi*, *Dactylogyrus vastator*, *Gyrodactylus salaris,* and *Ancyrocephalus*. The diversity of parasites was maximum at the Okara site. The host’s organs that were targeted for parasitic infection included the intestine, liver, gills, fins, skin, and kidneys. The majority of the parasites were identified in *Labeo rohita* followed by *Hypophthalmichthys molitrix*, *Ctenopharyngodon idella*, *Oreochromis niloticus*, *Cyprinus carpio*, and *Wallagu attu*. Two species appeared to be resistant species because none of the parasites were observed in *Notopterus notopterus* or *Sperata seenghala*. This study also concluded that the prevalence of parasites increased with increasing length, size, and age of fish.

## 1. Introduction

Disease outbreaks in fish are the most devastating challenge for aquaculture production. Many freshwater fish species are seriously afflicted with various parasites, which results in high fish mortality and reduced aquaculture productivity and has a negative influence on the economy [1]. Infections caused by numerous fish parasites can impede the development of a culture system. The significance of fish parasites is directly connected to the significance of fish health. The host’s tissues or digested gut contents are the primary sources of nutrition for fish parasites, and upon favorable conditions, the parasites reproduce rapidly [2].

The rate of parasitic infection in fish is high among vertebrates, which is related to the aquatic environment in terms of dispersion, life cycle, and reproduction [3]. Parasites are ubiquitous in Asian countries and thus reduce aquaculture productivity, which is an important source of employment in many countries [4]. Parasites enhance their transmission in fish by altering the host’s eating, mating, and social behavior and migration patterns [5]. They also influence the neurological system of their host, interfere with the secretary functions of the alimentary canal and metabolism, and damage fish skin and gills by causing sores, ulceration, and tissue deterioration [6]. Parasites can influence the structure of fish communities [7]. Adult parasites are more dangerous, depending on the parasite load and size of the host, as well as the form of attachment [8]. In certain circumstances, parasites do not kill fish, but they have a negative impact on the population and individual fish. Protozoans, trematodes, and monogeneans are the most common parasites of fish species [9].

Protozoan parasites are of great importance as they vary in size and shape. Protozoan parasites have diversified classification [10]. Among the protozoan parasites, *Ichthyobodo necator* (Henneguy, 1883), *Ichthyophthirius multifiliis* (Fouquet, 1876), *Trichodina* sp. (Ehrenberg 1831), and *Trichophyra* and *Hexamita* (J. R. Uzmann, J.W. Jesse 1963) are some of the most significant pathogens that cause diseases in aquaculture [11]. They raise farm inputs due to increased handling costs and yield insufficient growth rates due to disease outbreaks [12]. Protozoan parasites cause severe diseases in freshwater fishes all over the world including ichtyobodiasis, coccidiosis, ichtyopthiariasis, and trichodiniasis [13]. The mortality rate of infected fish can reach almost 100% [14]. Parasitic diseases have gained much attention in research as fish consumption has increased in the last decade.

Fishbone trematodes cause serious infections in humans if they are consumed improperly [15]. Fish-borne trematodes affect the health of more than 40 million people in the world [4]. In 2005, 56.2 million people were infected with foodborne trematodiasis, including 7158 deaths [16]. Many farmers experience economic losses due to trematode parasites [17]. Trematodes belong to the phylum Platyhelminthes. Adult trematodes are obligatory parasites of many vertebrates. Trematodes complete their life cycle in four hosts [18,19]. Trematodes of the family Heterophyidae are intestinal trematodes. Their final hosts are mammals and birds. There are 22 species in the Heterophyidae family that cause infection in humans worldwide. Some previous studies reported that *Haplorchis pumilio* and *Centrocestus formosanus* were zoonotic species found in Sutchi catfish, but some unidentified species were also found, suggesting that there is a need for further investigation [20]. There is a need to record the distribution of parasites in relation to host size, season, and farm management [21]. The effects of the trematode metacercariae on fish include delayed growth of young fish and decreased immunity of fish, due to which secondary infections may also occur. Some other diseases include black spot disease, malformations in fish, inflammation of the liver, necrotic tissue change, displacement of organs, functional morbidity, and severe gill damage [22]. 

Monogeneans are a typically diverse group of ectoparasites of freshwater and marine fishes [23]. Monogeneans worms and their communities in cultured fish affect ecosystem health [24]. Monogeneans cause an increase in fish mortality due to various infections such as respiratory problems, anemia, and osmoregulatory dysfunction, and they also cause secondary microbial infection. Mongenean infestations in fish aquaculture bring about large financial losses [25]. The monogenean life cycle was found to rapidly increase in an artificial environment, which caused injurious infection in their hosts [26]. Depending on the fish species, monogeneans attach to the gills, the surface of the skin, fins, and eyes and typically fed on the blood, mucus, and epidermal cells of their host. Small- to medium-sized monogenean parasites complete their life cycle in a single host [27]. Different species of monogenean have been involved in the death of wild and cultured fish such as tilapia [28]. Members of Dactylogyridea, Ancyrocephalidae, and Gyrodactylidae have been reported in cultured and wild fish. Transmission of these parasites mostly depends on host-to-host interactions, although parasites may also occupy a new host by drifting with water currents or depending on water quality, which directly affects their infection processes [29]. In polluted water, parasitic infections commonly increase, and they provide an indication of water quality [30]. 

Relatively little research has been conducted on freshwater fish parasites. The identification of parasites is important for determining the specific etiology of sicknesses. Once the diversity of fish parasites is established, identification of the disease-causing agent and their pathogenicity will be easily accessible. After the identification of parasites, the risk of infection can be determined using the prevalence of parasites in fish and different water bodies. The present study was therefore designed to identify parasites of freshwater fishes in River Ravi, Pakistan

## 2. Materials and Methods

### 2.1. Study Site

Fish samples were collected from 3 different harvesting sites (River Ravi Downstream Head Balloki District Kasur, Lower Bari Doab Canal District Kasur, and River Ravi Tehsil Okara) in River Ravi. Samples were collected during the harvesting season (2020–2021).


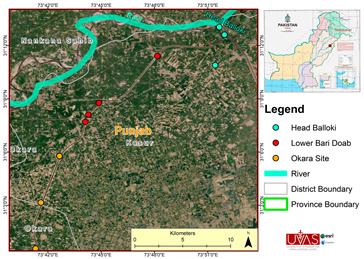


### 2.2. Sample Collection

In total, 405 fish samples (45 of each fish species) were collected using a gillnet to investigate the prevalence of infection, mean intensity, and abundance of protozoan, monogenean, and trematode parasites. The examined fishes were *Labeo rohita* (F. Hamilton, 1822), *Hypophthalmichthys molitrix* (Valenciennes, 1844), *Ctenopharyngodon idella* (Valenciennes, 1844), *Cirrhinus mrigala* (Hamilton, 1822), *Cyprinus carpio* (C. Linnaeus, 1758), *Wallagu attu* (Bloch and Schneider, 1801), *Notopterus notopterus* (Pallas, 1769), *Sperata seenghala* (Sykes, 1839), and *Oreochromis niloticus* (Linnaeus, 1758). The samples were examined physically to assess the general health status of the fish. For further investigation, fish samples were kept in plastic bags within ice boxes after labeling and then transported immediately to the Laboratory of Fisheries and Aquaculture, University of Veterinary and Animal Sciences, Ravi campus. 

### 2.3. Examination of Protozoan Parasites

#### 2.3.1. Study of External Protozoan Parasites

**Figure 1:**
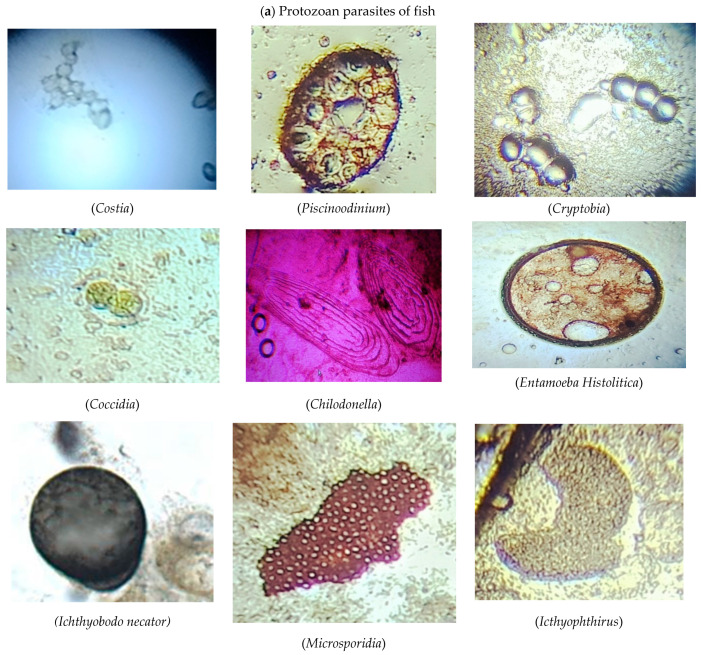

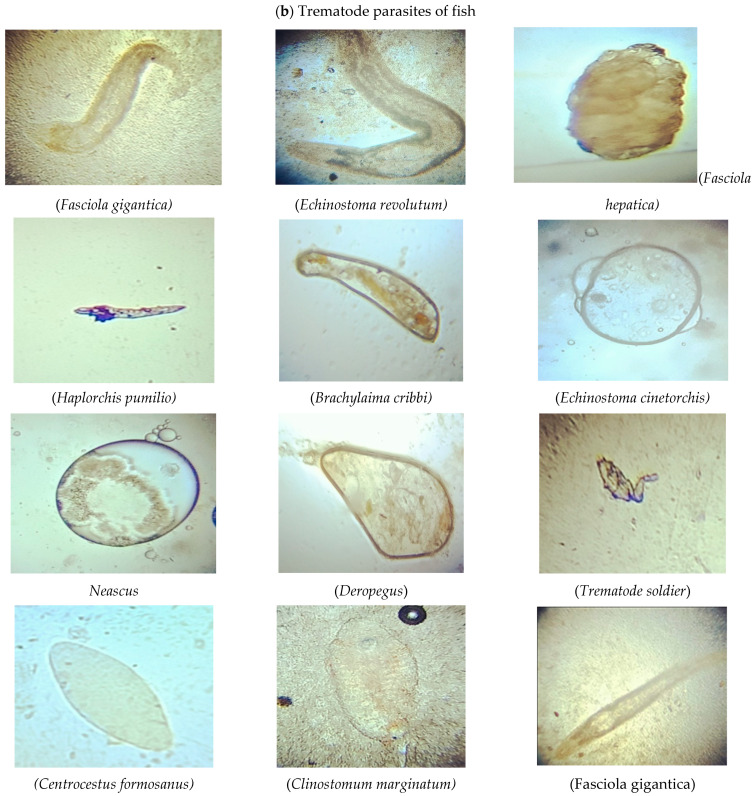

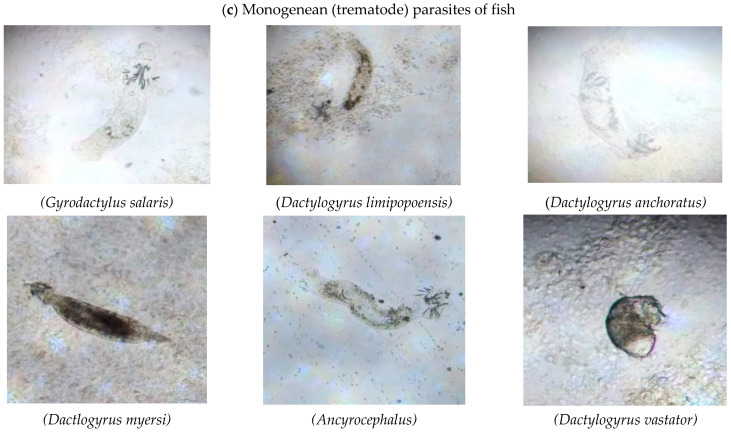
(**a**) Photographs of protozoan parasites along with their names, which were identified (Table 1) in the freshwater fish from River Ravi. (**b**) Photographs and names of trematode parasites that were identified in the freshwater fish from River Ravi. (**c**) Photographs and names of monogenean parasites that were identified in the freshwater fish from the three studied sites in River Ravi.

The length and weight of all the fish were recorded. For the study of ectoparasites, external organs of the fish such as skin, scales, fins, tails, etc., were examined with a hand lens. Permanent and wet mounts were prepared by scraping the dorsolateral surface of a fish with the dull side of a scalpel blade. Scrapings were obtained from near the base of all the fins [31]. To observe gill parasites, the operculum was removed from the fish. In the case of a small fish, the entire gill arch was removed, transferred to a slide, and examined under a microscope. In the case of a larger fish, the bony arch was also removed and examined under a stereomicroscope. Furthermore, the gills were also dissected from the branchial cavity and placed in a mixture of 1:4000 formalin solution for one hour. The decanted sediments were placed in a Petri dish and analyzed for protozoan parasites under a stereoscopic microscope at low and high magnifications [32]. Photographs of the identified external protozoan parasites are shown in Figure 1a.

#### 2.3.2. Study of Internal Protozoan Parasites

The fish were dissected for the detection of internal protozoan parasites in different organs (heart, liver, intestine, kidney, and spleen). Each organ was removed and separately bathed with a physiological saline solution (0.7% NaCl solution). Intestinal parasites were examined after placing 1–2 drops of intestinal fluid on microscopic slides. In the case of a small fish, the intestinal tract was opened along its entire length and examined under a stereomicroscope by compressing a longer section of the intestine between the glass slides. Wet mounts were prepared using a scraping from the affected fish after fixing it in a 10% buffered neutral formalin solution. These samples were then stained with hematoxylin and counter-stained with eosin for examination under a microscope at both low and high magnification [32]. Photographs of the identified internal protozoan parasites are shown in Figure 1a.

### 2.4. Examination of Trematode Parasites

#### 2.4.1. Detection of Metacercariae

Two general methods were used to examine the foodborne trematode (FBT) metacercariae in second intermediate hosts.

1. The muscle compression technique.
2. The pepsin-HCL artificial digestion technique.

#### 2.4.2. Muscle Compression

For this technique, samples were obtained from the muscles, intestine, kidney, heart, gills, and liver of the fish. Each sample was weighed to estimate the density of metacercariae. Each organ sample was compressed between two glass slides. Metacercariae were observed and identified under a stereo microscope. The number of metacercariae was counted and their density was calculated [33].

#### 2.4.3. Pepsin-HCL Artificial Digestion Technique

Samples of fish muscles, intestines, kidneys, heart, gills, and liver were ground one by one with a mortar and pestle. The ground samples were transferred to artificial gastric juice {Conc. HCl (10 mL) + pepsin (10 g) + distilled water (1000 mL)}, mixed well, and incubated at 37 °C for two hours with occasional stirring and filtering. Saline (0.85%) was added, the supernatant was thrown away carefully, and the sediments were again washed until the supernatant became clear. A small amount of sediment was transferred to a Petri dish in which physiological saline (6–7 mL) was present. A stereomicroscope and light microscope were used to count and identify the metacercariae on the basis of the shape of cysts, size of suckers, and shape of the excretory bladder, according to [34,35,36]. Photographs of the identified trematode parasites are shown in Figure 1b.

### 2.5. Examination of Monogenean Trematodes

A magnifying lens was used to check monogenean parasites on the external surface of fins, skin, and gills. Scalpel blades were used to obtain scrapings from the gills, skin, and fins for the examination of attached parasites. These scrapings were transferred to a clean microscopic slide to prepare wet mounts, which were examined under a stereomicroscope. From each gill filament, the gill arches were dissected using surgical scissors and then placed in Petri dishes for microscopic examination. Similarly, from the base of each fin, fin pieces were placed on slides, covered with coverslips, and then observed under a binocular microscope. Monogenean parasites were identified using sclerotized structures (anchors, transverse bar, vestigial ventral bar, hooks, and male copulatory organ) [31]. Photographs of the identified monogenean parasites are shown in Figure 1c.

### 2.6. Identification of Parasites

The identification of the examined protozoan, monogenean, and trematode parasite was completed using the standard keys [9,31,33,34,35,36,37].

### 2.7. Prevalence, Intensity, and Density of Parasites

The following formulas were used to estimate the prevalence, density, and intensity of parasites by following [38]:

Prevalence=no.of individual of a host infected with a particular parasite species÷no.of hosts examined×100

Intensity=sum of individuals of a particular parasite species in a sample of a host÷total of infected individuals of the host in the sample.

Density=sum of individuals of a particular parasite species in a sample of hosts÷total no.of individuals of host (infected+uninfected) in sample.

### 2.8. Statistical Analysis

Statistical analyses of the collected data were performed using statistical package for social sciences (SPSS) version 21.0. The chi-square test was used to compare the infection rate of parasites at different sites. A *p*-value < 0.05 was considered statistically significant for all analyses [39].

## 3. Results

During the harvesting season (2020–2021), nine species of freshwater fish were sampled from the three study sites. In total, 405 freshwater fish from these nine fish species (45 samples per fish species) were examined, including carp, catfish, and tilapia. The weight of the fish that were examined during this study was between 100 g and 3000 g. The results revealed that parasite prevalence was high in large-sized fish compared to small- or medium-sized fish.

Table 1: In total, 9 species of protozoan parasites, 11 species of trematodes, and six species of monogenean parasites were identified in different organs from seven freshwater fish species. Protozoan parasites were observed on the external and internal organs of fish such as the intestine, liver, kidneys, skin, and fins. Trematodes were found only in the intestine and gills. Monogeneans were found only on the external organs of fish, i.e., the skin, gills, and fins. Two fish species, *Sperata seenghala* and *Notopterus notopterus*, were identified as resistant species as none of the parasites was observed in either of these fish species.

Table 2: In total, 573 parasites were identified in 405 fish consisting of 210 protozoan parasites, 185 trematode parasites, and 178 monogenean parasites. The rate of parasite infection was calculated by counting the total number of parasite species for each class at a specific site. The chi-square (Χ^2^) statistic was used to analyze the relationship between parasites and a specific site. The level of significance was set at *p* < 5% (Table 2). The statistical analysis revealed that there was not any association between parasites and a specific site. The existence of parasites was not site-specific.

Table 3 lists the infection rate of individual fish species for each protozoan parasite including *Chilodonella, Coccidia, Costia, Cryptobia, Entamoeba histolitica, Icthyophthirus multifillus, Microsporidia, Piscinoodinium,* and *Ichthyobodo necator*. The number of infected fish with a certain number of protozoan parasites is also listed. The prevalence of infection, mean intensity, and mean abundance were calculated using the formulas mentioned in Section 2.6. Photographs of each protozoan parasite are shown with their names in Figure 1a.

Table 4 lists the infection rate of individual fish species for each trematode parasite including *Brachylaima cribbi, Centrocestus formosanus, Clinostomum marginatum, Deropegus* sp., *Echinostoma cinetorchis, Echinostoma revolutum, Fasciola hepatica, Fasciola gigantica, Haplorchis pumilio, Neascus*, and *Trematode Soldier*. The number of infected fish with a certain number of trematode parasites is also listed. The prevalence of infection, mean intensity, and mean abundance were calculated using the formulas mentioned in Section 2.6. Photographs of each trematode parasite are shown with their names in Figure 1b.

Table 5 lists the infection rate of individual fish species for each monogenean parasite including *Ancyrocephalus* sp., *Dactylogyrus* sp., *Gyrodactylus* sp. The number of infected fish with a certain number of monogenean parasites is also listed. The prevalence of infection, mean intensity, and mean abundance were calculated using the formulas mentioned in Section 2.6. Photographs of each monogenean parasite are shown with their names in Figure 1c.

## 4. Discussion

The significance of continuous surveillance of foodborne parasites and their epidemiological dispersion cannot be overstated in developing countries. Parasites cause pathogenic effects and financial damage to fish farming. The data in this study indicated the distribution of parasites in freshwater fish that were collected from three study sites along River Ravi (shown on the map). The fish diagnosed for parasite infestation were *Labeo rohita*, *Hypophthalmichthys molitrix*, *Ctenopharyngodon idella*, *Cirrhinus mrigala*, *Cyprinus carpio*, *Wallagu attu*, *Notopterus notopterus*, *Sperata seenghala*, and *Oreochromis niloticus*. During the fish investigation, nine protozoan parasites, 11 trematode parasites, and 6 monogenean parasites were observed under the microscope. A similar study was conducted in Turkey on the host–parasite relationship, which examined protozoa (14 species), monogenean (12), and trematode (15) parasites and some other taxa [11]. Similarly, another investigation was carried out in Bangladesh on the prevalence of protozoan and monogenean parasites in fish (*H. molitrix*, *C. idella*, C. *carpio*, *B. gonionotus*, *C. catla*, *L. rohita* and *C. cirrhosis*) by [1], and two species of protozoan parasites (Trichodina and Chilodonella) and one species of monogenean parasites (Dactylogyrus) were examined. The results of the preSSsent study showed some dissimilarities with [1] because Trichodina was not observed in any fish, and some other parasites were also identified in our study. Another study was performed by [40] on large-sized silver and common carp. Their findings identified two protozoans (Trichodina and Ichthyophthirius multifiliis), two monogeneans (Dactylogyrus and Gyrodactylus), and two crustacean parasites, which is pertinent to this study because the majority of parasites were seen in large-sized fish. The photographs of parasites in Figure 1 were similar to the findings of [9,31,33,35,36,37,38]. In this study, the majority of parasites were found in *L. rohita*, followed by *H. molitrix*, *C. idella*, *O. niloticus*, *C. carpio*, and *W. attu*.

This study examined protozoan parasites including *Coccidia, Piscinoodinium, Microsporidia, Icthyophthirus multifillus, Costia, Ichthyobodo necator, Cryptobia, Chilodonella,* and *Entamoeba histolitica*. The prevalence of infection, mean intensity, and abundance of protozoan parasites that infected the fish is mentioned in Table 3. In [41], an investigation was carried out on 11 protozoan parasites that had infected *Clarias gariepinus*. Out of the eleven protozoan parasites, four parasites were also identified in this study, including *Piscinoodinium, Coccidia, Chilodonella,* and *Microsporidians*. Another protozoan parasite, Costia, was also identified in the findings of [13,41]. In [42,43,44,45,46,47,48], it was reported that *Trichodina* and *Ichthyophthirius multifiliis* were the prevalent protozoan parasites on the skin and gills of cyprinid fish. In the present study, Ichthyophthirius was observed in rohu and silver carp. In [49], the authors described chronic infections of catfish by the protozoan parasite Ichthyophthirius on skin and gills, Ichthyobodo and Chilodonella on the skin, and Cryptobia in the stomach and intestine. As compared to [49], the present study identified that Ichthyophthirius was present in intestine, Chilodonella was identified on skin, and Cryptobia was identified in the intestine. In [11], the authors identified harmful protozoan parasites and their infections, such as Trichodina, Tetrahymena, Ichthyophthirius, and Ichthyobodo necator. This work showed closeness with the current study because the protozoan parasite Ichthyophthirius and its prevalence was identified in different fish species. Our results relate to the findings of [50], which found most protozoan parasites in the intestine. A decline in protozoan parasites in the stomach was found to be due to the acidic nature of the stomach because protozoan parasites occupy a specific pH medium [51,52].

This study examined monogenean parasites including *Dactylogyrus limipopoensis*, *Dactylogyrus anchoratus*, *Dactylogyrus myersi*, *Dactylogyrus vastator*, *Gyrodactylus salaris*, and *Ancyrocephalus* (mentioned in Table 3). The highest prevalence of parasites was observed in *Labeo rohita*. *Gyrodactylus salaris* was first discovered in farmed salmonids in Romania [53,54]. In the present study, *Gyrodactylus* was observed in carp. According to [47], 70 species of Dactylogurus were reported in both wild and farmed common carp in Iran. In the present study, four species of Dactylogyrous (*D. limipopoensis, D. anchoratus, D. myersi* and *D. vastator*) were observed in the following freshwater fish from River Ravi: *Hypophthalmichthys molitrix, Labeo rohita, Cyprinus carpio*, and *Oreochromis niloticus*. A study by [44] reported that *D. anchoratus* and *D. extensus* were observed within a specific range of water quality parameters. Furthermore, [55] described that infection with a monogenean parasite could be dangerous and harmful for fries in hatcheries. The results of the present study also coincide with the findings of [40], which reported that two monogeneans, *Dactylogyrus* ssp. and *Gyrodactylus*, were observed in *H. molitrix* and *C. carpio*. These results are in conformance with [46], who investigated *Dactylogyrus* sp. and *Gyrodactylus* in *Channa pleurophtalma* in terms of their dominance on the gills.

This study examined trematode parasites including *Fasciola hepatica, Trematode Soldier, Haplorchis pumilio, Brachylaima cribbi, Echinostoma cinetorchis, Clinostomum marginatum*, *Deropegus* sp., *Neascus* sp., *Fasciola gigantica, Echinostoma revolutum, and Centrocestus formosanus*. A microscopic examination of endoparasites of commercially important fish from Egypt was performed by El-shahawy, in which only one trematode and two cestodes were identified, while in the present study, none of the cestodes was observed. A study by [35] identified the infection of many fish species with metacercariae from four species of trematodes. In the present study, two trematode parasites were identified in freshwater fish that resemble the findings of [35], including *Haplorchis pumilio* in the intestine of grass carp [56] and *Centrocestus formosanus* in the intestine of silver carp [57]. *Haplorchis pumilio* was detected in the intestine of grass carp from the Lower Bari Doab Canal. Thien et al. (2009) analyzed the same parasites in catfish. Metacarcaria of *C. formosanus* were also observed in fish during a study by [58,59,60]. Two species belonging to two genera of zoonotic trematode parasites were recorded with different prevalence rates (*Centrocestus formosanus, Centrocestus sinensis, Haplorchis taichui,* and *Haplorchis pumilio*) in various freshwater fish species from local markets in northern Vietnam [61], while in the present study, only one species from both genera was observed. Two trematode parasites, *Clinostomum marginatum* and *Neascus* sp., observed in the present study also relate to the findings of [62]. In the present study, it was also found that large-sized fish had more parasites than smaller fish. This parasitic load in bigger fish was suggested to result from being exposed to a variety of parasites while foraging for food [42]. It was also found that the majority of parasites was observed at the Okara site. 

## 5. Conclusions

A wide diversity of protozoan, trematode, and monogenean parasites was observed in different organs of freshwater fish species. The highest burden was of protozoans in carps, which can cause serious detrimental effects on fish health and the economy. Documentation of these parasites is important to determine their ecological role and the economic value of the losses they cause in natural waters. The identification of fish parasites and their density and diversity is very important to correctly determine the infectious agents and to assess the safety techniques used to improve natural fauna and flora in better way. 

## Figures and Tables

**Table 1:** Parasites and their infection sites in the fish species obtained from different harvesting localities in River Ravi.

Identified Parasites	Host	Infection Site	Locality
Protozoans			
*Microsporidia* (Balbiani, 1882)	*Labeo rohita*	Liver	LBD Canal
*Icthyophthirus multifillus* (Foquet, 1876)		Intestine	
*Costia* (Henneguy, 1883)	*Cirrhinus mrigala*	Kidney	
*Ichthyobodo necator* (Henneguy, 1883)	*Cyprinus carpio*	Liver	
*Cryptobia* (E. Nohynkova, 1984)	*Labeo rohita*	Intestine	Head Balloki
*Chilodonella* (Kiernik, 1909)		Skin	
*Entamoeba histolitica* (Chatton, 1909)	*Ctenopharyngodon idella*	Liver	
*Coccidia* (WT. Johnson, 1892)	*Labeo rohita*	Gills	Okara
*Ichthyophithirus*	*Hypophthalmichthys molitrix*	Intestine	
*Chilodonella*		Skin	
*Piscinoodinium* (Schaperclaus, 1954)		Intestine	
*Entamoeba histolitica*	*Wallagu attu*	Liver	
Trematodes			
*Fasciola hepatica* (Linnaeus, 1758)	*Labeo rohita*	Gills	LBD Canal
Trematode Soldier (Gibson, 1996)		Intestine	
*Haplorchis pumilio* (Looss, 1896)	*Ctenopharyngodon idella*	Intestine	
*Brachylaima cribbi* (A.R. Butcher, 2003)			
*Echinostoma cinetorchis* (Ando & Ozaki, 1923)	*Labeo rohita*	Gills	Head Balloki
*Clinostomum marginatum* (Rudolphi, 1819)	*Hypophthalmichthys molitrix*	Intestine	
*Deropegus* sp. (McCauley, 1961)	*Ctenopharyngodon idella*	Intestine	
Neascus (Hoffman, 1955)			
*Fasciola gigantica* (Cobbold, 1853)	*Labeo rohita*	Gills	Okara
*Echinostoma revolutum* (Frohlich, 1802)			
*Centrocestus formosanus* (Nishigori, 1924)	*Hypophthalmichthys molitrix*	Intestine	
Monogeneans			
*Gyrodactylus* (Malmberg, 1957)	*Ctenopharyngodon idella*	Gills	LBD Canal
*Dactylogyrus* (Kulwiec, 1927)	*Hypophthalmichthys molitrix*	Fins	
*Gyrodactylus*	*Oreochromis niloticus*	Skin	
*Dactylogyrus*	*Labeo rohita*	Skin	Head Balloki
*Dactylogyrus*		Fin	
*Ancyrocephalus* (Creplin, 1839)	*Hypophthalmichthys molitrix*	Gills	
*Gyrodactylus*	*Cyprinus carpio*	Gills	Okara
*Dactylogyrus*		Skin	
*Ancyrocephalus*	*Ctenopharyngodon idella*	Gills	
*Dactylogyrus*	*Oreochromis niloticus*	Skin	

**Table 2:** The rate of parasite infection at three different study sites in River Ravi.

Study Site	Observed Parasites	Number	Infection Rate	Χ^2^	*p*-Value
Protozoans					
LBD	*Microsporidia*	16	36%	16.333	0.569
	*Icthyophthirus multifillus*	12			
	*Costia*	20			
	*Ichthyobodo necator*	28			
Head Balloki	*Cryptobia*	16	21%		
	*Chilodonella*	18			
	*Entamoeba histolitica*	10			
Okara	*Coccidia*	20	43%		
	*Ichthyophithirus*	10			
	*Chilodonella*	20			
	*Piscinoodinium*	32			
	*Entamoeba histolitica*	8			
Treematodes					
LBD	*Fasciola hepatica*	15	34.5%	22	0.341
	Trematode Soldier	20			
	*Haplorchis pumilio*	12			
	*Brachylaima cribbi*	17			
Head Balloki	*Echinostoma cinetorchis*	20	30%		
	*Clinostomum marginatum*	12			
	*Deropegus* sp.	16			
	Neascus	8			
Okara	*Fasciola gigantica*	23	35%		
	*Echinostoma revolutum*	30			
	*Centrocestus formosanus*	12			
Monogeneans					
LBD	*Gyrodactylus*	30	32.5%	23	0.310
	*Dactylogyrus*	28			
Head balloki	*Dactylogyrus*	20	25%		
	*Ancyrocephalus*	25			
Okara	*Gyrodactylus*	20	42%		
	*Dactylogyrus*	27			
	*Ancyrocephalus*	28			

**Table 3:** Infection rate of each fish species for each protozoan parasite.

Parasite	Host (n = 45/Species)	Infected Fish (%)	Parasite Number (N)	Prevalence of Infection (*p*)	Mean Intensity (MI)	Mean Abundance (MA)
*Chilodonella*	*Labeo rohita*	10 (0.22)	18	22.2	1.8	0.4
	*Hypophthalmichthys molitrix*	8 (0.17)	20	17.6	2.5	0.4
*Coccidia*	*Labeo rohita*	15 (0.33)	20	33.2	1.33	0.4
*Costia*	*Cirrhinus mrigala*	19 (0.42)	20	42.2	1.05	0.4
*Cryptobia*	*Labeo rohita*	10 (0.22)	16	22.2	1.6	0.35
*Entamoeba histolitica*	*Ctenopharyngodon idella*	10 (0.22)	10	22.2	1	0.22
	*Wallagu attu*	4 (0.08)	8	48.8	2	0.17
*Icthyophthirus multifillus*	*Labeo rohita*	15 (0.33)	12	33.2	0.8	0.26
	*Hypophthalmichthys molitrix*	5 (0.11)	10	11	2	0.22
*Microsporidia*	*Labeo rohita*	12 (0.26)	16	26.9	1.3	0.35
*Piscinoodinium*	*Hypophthalmichthys molitrix*	25 (0.55)	32	55.4	1.28	0.71
*Ichthyobodo necator*	*Cyprinus carpio*	18 (0.4)	28	40	1.55	0.62

**Table 4:** Infection rate of each fish species for each trematode parasite.

Parasite	Host (n = 45/Species)	Infected Fish (%)	Parasite Number (N)	Prevalence of Infection (*p*)	Mean Intensity (MI)	Mean Abundance (MA)
*Brachylaima cribbi*	*Ctenopharyngodon idella*	12 (0.26)	17	26.6	1.41	0.36
*Centrocestus formosanus*	*Hypophthalmichthys molitrix*	10 (0.22)	12	22.2	1.2	0.26
*Clinostomum marginatum*	*Hypophthalmichthys molitrix*	11 (0.24)	12	24.4	1.09	0.26
*Deropegus* sp.	*Ctenopharyngodon idella*	14 (0.31)	16	31	1.14	0.34
*Echinostoma cinetorchis*	*Labeo rohita*	18 (0.4)	20	40	1.11	0.44
*Echinostoma revolutum*	*Labeo rohita*	26 (0.57)	30	57.6	1.15	0.66
*Fasciola hepatica*	*Labeo rohita*	12 (0.26)	15	26.6	1.25	0.32
*Fasciola gigantica*	*Labeo rohita*	19 (0.42)	23	42.2	1.21	0.5
*Haplorchis pumilio*	*Ctenopharyngodon idella*	10 (0.22)	12	22.2	1.2	0.26
Neascus	*Ctenopharyngodon idella*	7 (0.15)	8	15.4	1.14	0.16
Trematode Soldier	*Labeo rohita*	15 (0.33)	20	33.2	1.33	0.44

**Table 5:** Infection rate of each fish species for each monogenean parasite.

Parasite	Host (n = 45/Species)	Infected Fish (%)	Parasite Number (N)	Prevalence of Infection (*p*)	Mean Intensity (MI)	Mean Abundance (MA)
*Gyrodactylus*	*Ctenopharyngodon idella*	15 (0.33)	12	33.3	0.8	0.26
*Ancyrocephalus*		15 (0.33)	10	33.3	0.6	0.22
*Dactylogyrus*	*Hypophthalmichthys molitrix*	38 (0.84)	24	84.4	0.63	0.53
*Ancyrocephalus*		38 (0.84)	20	84.4	0.5	0.44
*Dactylogyrus*	*Labeo rohitaza*	40 (0.88)	46	88.8	1.15	1
*Gyrodactylus*	*Cyprinus carpio*	23 (0.51)	15	51	0.65	0.33
*Dactylogyrus*		23 (0.51)	13	51	0.5	0.28
*Gyrodactylus*	*Oreochromis* *Niloticus*	15 (0.33)	10	33.3	0.6	0.22
*Dactylogyrus*		15 (0.33)	8	33.3	0.53	0.17

## Data Availability

Not applicable.
